# *A posteriori* correction of camera characteristics from large image data sets

**DOI:** 10.1038/srep10317

**Published:** 2015-06-11

**Authors:** Pavel Afanasyev, Raimond B. G. Ravelli, Rishi Matadeen, Sacha De Carlo, Gijs van Duinen, Bart Alewijnse, Peter J. Peters, Jan-Pieter Abrahams, Rodrigo V. Portugal, Michael Schatz, Marin van Heel

**Affiliations:** 1 Leiden Institute of Chemistry, Leiden University, 2333 CC Leiden, The Netherlands; 2 The Institute of Nanoscopy, Maastricht University, 6211 LK Maastricht, The Netherlands; 3 Netherlands Centre for Electron Nanoscopy (NeCEN), 2333 CC Leiden, The Netherlands; 4 FEI Company, 5651 GG Eindhoven, The Netherlands; 5 Brazilian Nanotechnology National Laboratory – LNNano, CNPEM, C.P. 6192, 13083-970 Campinas SP, Brasil; 6 Image Science Software GmbH, Gillweg 3, D-14193 Berlin, Germany; 7 Faculty of Natural Sciences, Imperial College London, London SW7 2AZ, UK

## Abstract

Large datasets are emerging in many fields of image processing including: electron microscopy, light microscopy, medical X-ray imaging, astronomy, etc. Novel computer-controlled instrumentation facilitates the collection of very large datasets containing thousands of individual digital images. In single-particle cryogenic electron microscopy (“cryo-EM”), for example, large datasets are required for achieving quasi-atomic resolution structures of biological complexes. Based on the collected data alone, large datasets allow us to precisely determine the statistical properties of the imaging sensor on a pixel-by-pixel basis, independent of any “*a priori*” normalization routinely applied to the raw image data during collection (“flat field correction”). Our straightforward “*a posteriori*” correction yields clean linear images as can be verified by Fourier Ring Correlation (FRC), illustrating the statistical independence of the corrected images over all spatial frequencies. The image sensor characteristics can also be measured continuously and used for correcting upcoming images.

In recent years, digital image data acquisition with image transducers like CCD/CMOS chips^1^ has become the standard, superseding the earlier analogue imaging technologies. Digital image transducers contain millions of pixels, each having (slightly) different characteristics in response to identical input signals. Indeed, “dead” pixels (or rows, columns, spots of pixels) may not give any response at all. In contrast, “hot” pixels may always produce a strong output independent of the input signal. The image presented to the user is typically a corrected image in which such pixels have been replaced by averages of neighbouring pixels, rows, or columns. The sensitivity differences between pixels will also be affected by flat-field correction^2^. We will call all such pre-processing the “*a priori*” correction associated with the image transducer/sensor.

The precision required for the correction of a set of images depends on their intended use. For example, in standard digital photography each image is appreciated individually, and the image sensor flaws after the *a priori* correction are normally not discernible. This means that the image errors are small compared to the 6-12 bits dynamic range of the red, green, and blue channel image information. The situation changes when the image has only very small contrast variations (such as an image of a homogeneous white wall) especially when dirt has accumulated on the sensor surface, effectively changing its properties compared to when the *a priori* correction parameters were determined. In such cases, flaws in an individual image can be directly visible.

When thousands or tens-of-thousands of images are to be studied extensively by advanced averaging algorithms, a simple *a priori* correction can prove insufficient. In the total average of a 10,000-images dataset, for example, the contrast of a fixed-pattern background image increases 10,000-fold since this small but fixed pattern adds up coherently. At the same time, the contrast due to actual image information – uncorrelated from frame to frame – increases by only a factor 100 (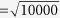). The ratio of the residual fixed-pattern variance, over the variance of the summed image information, thus increases 10,000 fold in the averaging process. A typical example is shown in Fig. 1a-c where ~10,000 cryo-EM images are averaged. The correct representation of the data in quantitative scientific image acquisition can thus be critical, depending on intended use.

The development of advanced digital cameras has been instrumental in pushing the resolution attainable by single-particle cryo-EM towards near-atomic levels^3^,^4^,^5^,^6^,^7^,^8^. This approach requires the use of large datasets to bring the noisy, low-contrast image information in the micrographs to statistical significance through extensive averaging procedures. In cryo-EM, the calibration of the *a priori* correction must be repeated regularly and performed under approximately the same conditions used in the subsequent data collection, since the pixel properties may change with the average exposure level^5^. Movie-mode data collection procedures require alignments based on correlation functions of images collected with the same sensor^5^,^9^. Insufficiently corrected images can lead to alignments with respect to the spurious zero-image of the chip rather than to the information content of the individual image frames.

When a large digital image dataset is available, collected with the same image transducer, then that dataset itself can be used for the statistical characterization of every pixel in the sensor. Images from different parts of the sample are in principle uncorrelated; thus, when summing all images from such a large dataset, the image information averages out. What prevails is the systematic different response of the individual pixels to the same average exposure. Different pixels also exhibit a different sensitivity or “gain”: very sensitive pixels will exhibit a larger standard deviation from the average exposure than do less sensitive pixels. For characterizing each pixel in the transducer, we study the statistics of associated pixel vector: the collection of all density measurements from that pixel throughput the dataset^10^. We characterize each pixel in terms of the average density and standard deviation of its pixel vector and exploit that information to normalize its output.

## Results

### A Posteriori Image Dataset Correction

We assume that a reasonable *a priori* correction has taken place which includes the masking out of dead (or ‘hot’) pixels, column, rows etc. in the physical transducers thus avoiding “division-by-zero” problems. Our *a posteriori* correction can then have the simple form:


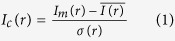


where the corrected image intensity 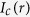 is derived from the measured raw image 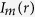 by subtracting 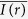, the average image over the full large dataset, normalized by the standard deviation image of that dataset 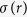 (dimensionless units are used throughout). The thus corrected images are normalized to zero mean and unit standard deviation per pixel vector throughout the dataset. The assumption behind this approach is that the input images used for the calculation of the average image 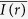 are uniform and indiscriminate in terms of the position of objects in the image.

### Examples

After the *a posteriori* data normalization, all pixel vectors will show the same statistical behaviour in that each pixel vector will have the same average density and the same standard deviation. As mentioned, camera manufacturers will typically replace dead (and hot) pixels by an average of surrounding pixels in their *a priori* correction. Thus, such flaws are normally not obvious in the average image 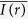. However, since the density provided for those poorly performing pixels is some average of their surroundings, the variance (or standard deviation) of the values found for that specific pixel vector will typically be lower than that of its fully functional peers. One can thus see these manipulated pixels/row/patches to behave differently in the 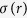 image derived from the full dataset.

**Figure 1:**
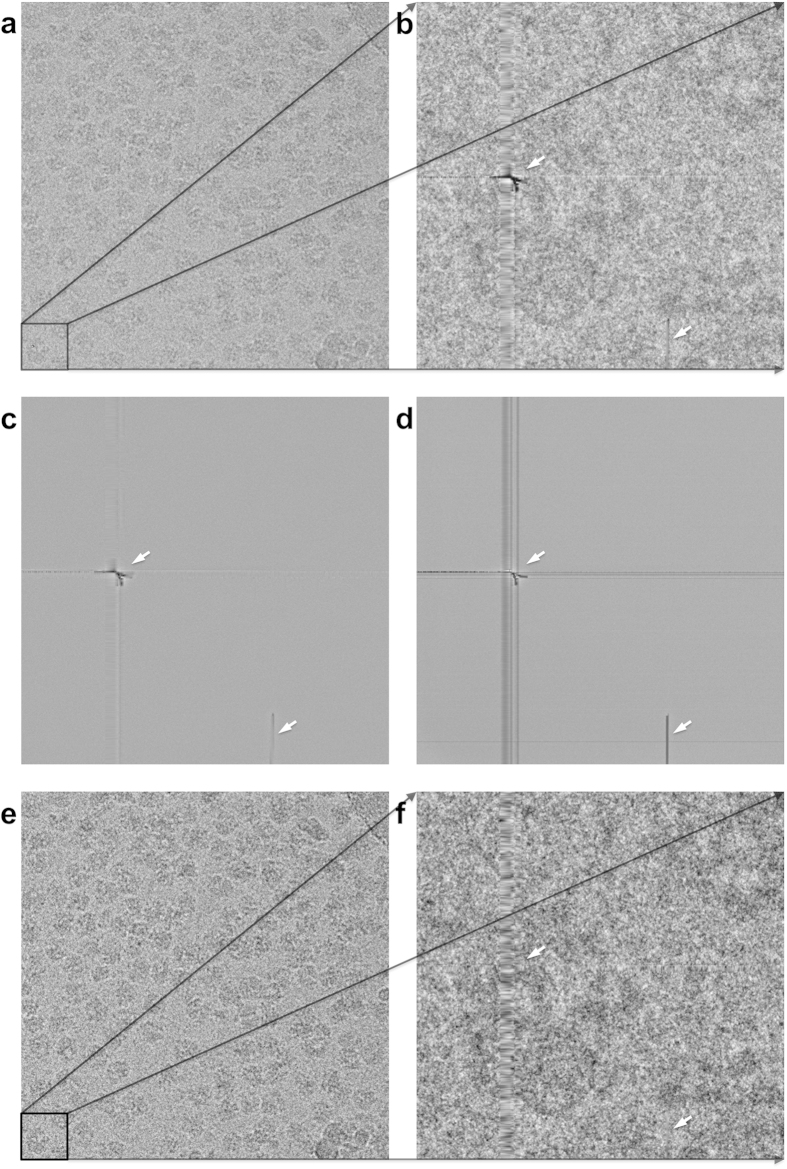
One full raw 4096 × 4096 ribosome image (**a**), and one specific zoomed-in 512 × 512 patch (lower-left corner) extracted from that image (**b**). This patch was the worst patch we could find in this experimental 4096 × 4096 back-thinned CMOS direct electron detection camera. The chip errors are such that they can be visually pinpointed in the image in spite of the *a priori* correction of the data. The average of all corresponding patch images from the full dataset – a total of 10821 images - is shown in frame (**c**). Spurious vertical and horizontal lines and other serious “fixed pattern” defects become clearly visible, while the ribosome images disappear altogether due to the averaging. The corresponding patch in the σ-image (**d**) reveals a strong bundle of about 16 vertical lines (marked by the left arrow) that were well suppressed by the standard *a priori* correction. While this suppression apparently included the averaging of pixel information in the immediate vicinity of these “dead” pixels and lines, the sensitivity of these chip areas collapses as is revealed by the dark areas in the σ-image. Moreover, the σ-image also reveals a thin horizontal line at the bottom of the patch (lower arrow) that had been corrected out in the average image (**c**), but again without compensating for the gain anomalies generated by the defect. The *a posteriori* corrected images are shown in panels (**e**) and (**f**) derived from the images shown in panels (**a**) and (**b**), respectively. Interestingly the *a posteriori* correction managed to improve on the dataset even by visual criteria although the more relevant metric is the FRC (see Fig. 2). The amplitude spectra of the average and standard-deviation images are shown in the Supplementary Fig. S1.

**Figure 2:**
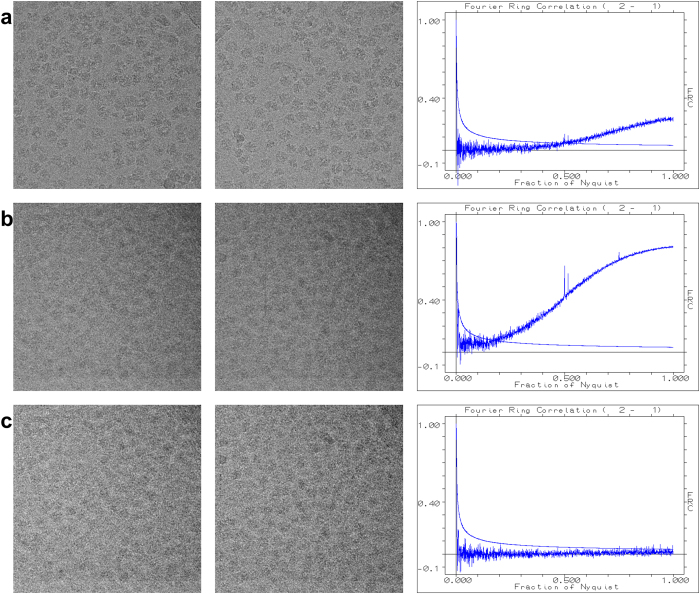
(**a**) Two different images collected on the same sensor (dataset of Fig. 1) can show a strong correlation of the fine image details (high frequency Fourier space components) due to a common background pattern in the image transducer. The FRC curve shows that the high-frequency information fully exceeds (by more than 3σ) the level of expected random-noise correlations. (**b**) This effect can be exaggerated if we first average a number of raw input images to yield two image averages (12 different images per average are used here) and only then to perform the FRC calculations between these two average images. The extra peaks at 0.5 and 0.75 of the Nyquist frequency in the FRC curve, are associated with fixed sensor readout patterns of the on-chip electronics. (**c**) The FRC of the same averaged image-sets illustrates that after the *a posteriori* correction the systematic background pattern is virtually removed from the data. Note that, in this 12-fold exaggerated critical test, the correction of the residual sensor pattern is close-to perfect.

In the cryo-EM example detailed in Figure 1, bad image areas can be discerned directly in the raw images (Fig. 1a,b) but especially in the average image (Fig. 1c) and the standard deviation image (σ-image, Fig. 1d). Whereas a set of vertical lines and some horizontal lines (marked by two arrows) are not very prominent in the overall average image (Fig. 1c) their “hiding” by averaging over an environment is clearly visible in the σ-image of the same sensor area (Fig. 1d). The *a posteriori* corrected images contain significantly less artefacts (Fig. 1e,f), however, the truly “dead” or “hot” pixel areas (like the columns and rows marked in Fig. 1e) cannot be fully corrected since they only repeat the information from neighbouring pixels and contain no new information. Spurious frequency-dependent correlations between different images are discussed below (Fig. 2).

**Figure 3:**
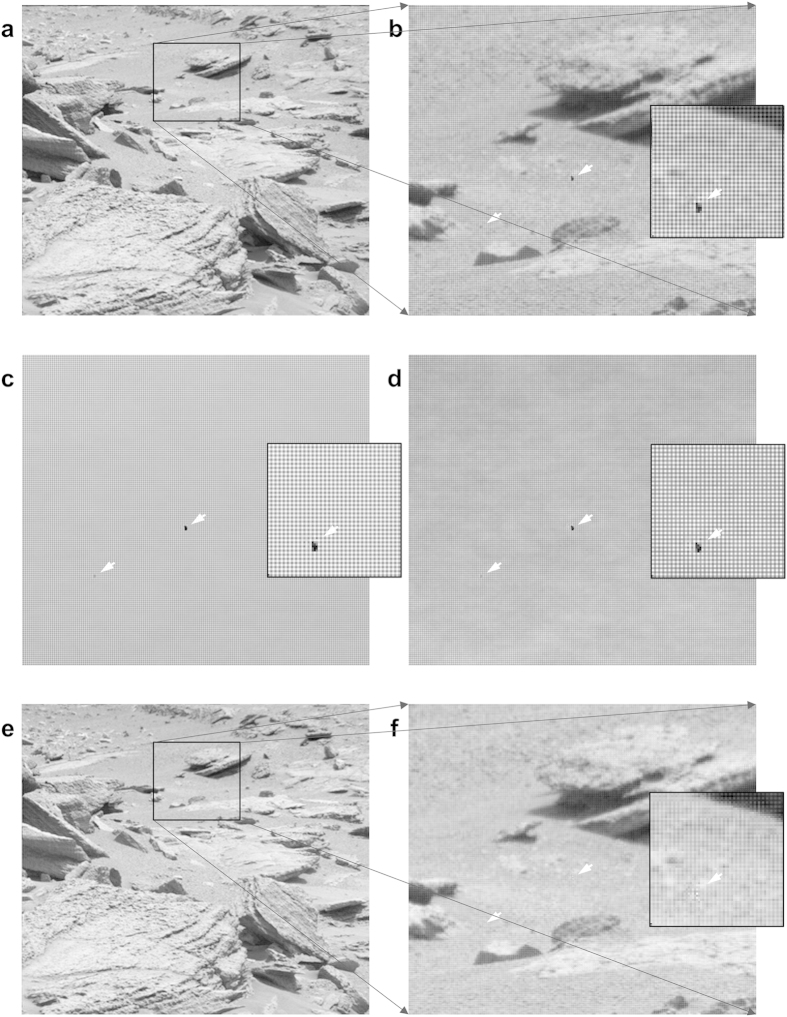
In (**a**) a single 1344 × 1200 pixel image, from the “Mastcam right” camera (MSSS-MALIN) of the NASA Mars rover Curiosity, is shown together with a 300 × 336 pixel detail (**b**). We used 1064 raw images of 1344 × 1200 pixels from this camera (http://mars.jpl.nasa.gov/msl/multimedia/raw/) to find the average image (see 300 × 336 detail (**c**); the central part of that is shown as an extra inset) and the σ-image image (300 × 336 detail: (**d**)). Apart from the strong visibility of the Bayer pattern in the average image of this camera, a block of 3 × 5 pixels with a very poor response is marked by a white arrow in the various “detail” images. A smaller anomaly visible in both the average - and the standard deviation image is marked by another white arrow. The *a posteriori* corrected image is shown in panel (**e**) and in detail in (**f**). The Bayer pattern is now largely invisible as are the other marked anomalies. The improvement of Fourier Ring Correlation between different images of this dataset by the *a posteriori* correction is discussed in Fig. 4.

As another example of the sensor correction, we use 1064 raw images of the Mars surface, collected by the Curiosity rover. These images of 1344 × 1200 pixels each (Fig. 3a) are from the Mastcam-right camera of the rover and are/were available from: http://mars.jpl.nasa.gov/msl/multimedia/raw/ [2 October 2014]). Their average (detail) is shown in Fig. 3c, and their σ-image image (detail) in Fig. 3d. The main purpose of the exercise was to correct for the different black-&-white sensitivities of the red-green-blue pixels of the “Bayer pattern” of the sensor in the raw images (before correction: Fig. 3a, b; after correction: Fig. 3e, f). Further anomalies of the sensor emerged (see legend of Fig. 3). Further examples of the procedure applied to data from different fields of science are given in the Supplementary Figures S1–S8.

### Validation by Fourier Ring Correlation

**Figure 4:**
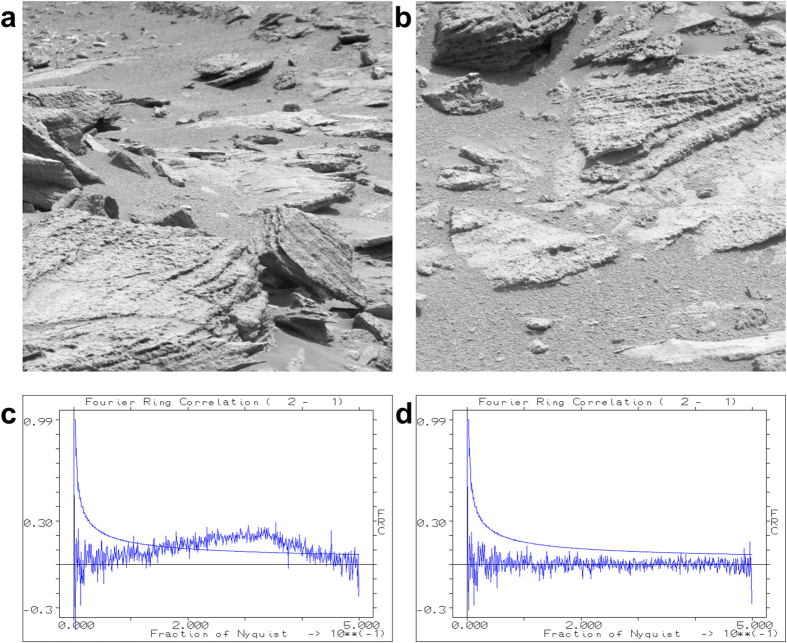
Fourier Ring Correlation (FRC) of two Mars rover images. Two typical images (**a** and **b**) taken from the Mastcam-right camera dataset (Fig. 3) are compared to each other by cross-correlation as function of spatial frequency (the central 1200 × 1200 pixels part of the images were used). The FRC of the raw uncorrected images (**c**) shows significant correlations (above 3σ of the theoretically expected value for random noise correlations) at around the 0.5 Nyquist frequency range, associated with the repeat of the 2 × 2 pixel Bayer pattern of the sensor. After *a posteriori* correction, the FRC oscillates around the zero value up to the Nyquist frequency (**d**).

To assess the quality of the *a posteriori* correction, we use the Fourier Ring Correlation (FRC) in which the normalized correlation coefficient is calculated between two different images over corresponding concentric rings in Fourier space^11^,^12^,^13^,^14^,^15^. This “gold standard” for assessing the reproducible resolution in two or three dimensions has recently also become popular outside the field of electron-microscopy^16^,^17^,^18^,^19^. Here, however, we use the full FRC curve to assess the *independence* of two images as function of spatial frequency (Fig. 2,4) rather than their cross resolution. The 3σ threshold curve indicates the maximum correlation levels expected between two independent random images. What is thus expected with a successful correction is an FRC curve that oscillates around zero and essentially never touches the positive (or negative) 3-sigma threshold curves^15^. The behaviour of the FRC close to the origin is not very relevant since that reflects just a few pixels in Fourier space and may suffer from fluctuations between the extreme values “−1” and “+1”.

When different object areas are imaged on the same part of the sensor, the FRC can show spurious correlations due to an insufficient *a priori* flat-field correction. In Fig. 2, examples are given for the FRC curve between two such cryo-EM images of ribosomes before and after the *a posteriori* correction. The FRC curve for this comparison exceeds the random-fluctuations threshold of 3σ expected for uncorrelated images. To better visualize this effect we also averaged two groups of twelve images of different object areas collected on the same area of the chip, to emphasize the influence of the fixed background pattern (Fig. 2b). The FRC peaks at 0.5 and 0.75 times the Nyquist frequency are due to systematic errors in the readout electronics of the camera. The “*a posteriori*” normalization corrects for the artificial correlations at high frequency as well as for the peaks at 0.5 and 0.75 times the Nyquist frequency (Fig. 2c). Note that, by averaging twelve frames, we emphasize the influence of a failing *a priori* correction; the *a posteriori* correction is nevertheless capable of suppressing the undesired background-pattern correlations in this critical test. Figure 4 illustrates the spurious correlations existing between two arbitrary images chosen from the Mars dataset before and after the *a posteriori* correction.

## Discussion

In the processing of digital images, *a priori* flat-field corrections are routinely applied to every collected raw image. Apart from measures to smoothen errors like dead and hot pixels, the typical procedures applied to normalize the behaviour of all pixels in a camera include the subtraction of a dark-image, and a pixel-by-pixel gain correction^2^,^9^. More elaborate corrections may include refinements to compensate for non-linearity of the pixel sensitivity^5^. As we have seen in most cases tested (see also the various sensors discussed in the Supplementary Information), however, the routine *a priori* dataset corrections are insufficient for many advanced data processing needs. Indeed, especially the σ-images derived from *a priori* corrected image datasets clearly reveal significant gain differences over the surface of the chip. Moreover, strong spurious Fourier-space correlations are revealed by FRC.

The proposed *a posteriori* correction aims at optimizing a full image dataset collected under similar conditions, based on the idea that all pixels should behave equally in a statistical sense. Small departures from an average signal on an individual pixel will always give a linear response on output. The *a posteriori* corrections will generally give clean linear results, irrespective of, for example, the average exposure level of the raw images. With a reasonable *a priori* correction in place, the linearity of the output data, after *a posteriori* correction, can extend over the full dynamic range of the image transducer.

An important development in single-particle cryo-EM is the introduction of movie-mode data collection. Here, rather than taking a single individual image of each area of the sample, a full sequence of individual “movie” frames is collected. The movie frames are then aligned relative to each other and then summed into a single overall average image^4^,^5^,^9^,^20^. Although the rationale is 30 years old^9^, three decades of instrumental and methodological developments have now brought the cryo-EM approach into the realm of atomic resolution^8^. Movie-mode data collection on direct electron detectors has contributed significantly to this development^4^,^5^. Critical is the aligning of the individual low-dose frames constituting a “movie”^4^,^5^,^9^,^21^ in producing the best possible image of the sample. Avoiding alignments of the movie frames to a fixed background patterns rather than to the actual image information is critical. Our *a posteriori* correction provides a new perspective for minimizing this problem.

The boundary between the *a priori* and *a posteriori* background correction is not always clearly defined. The individual pixel sensitivities can change over time or as a function of the ambient temperature^22^. One may thus want to apply the *a posteriori* parameters extracted from a similar recent data collection as an *a priori* correction to a new dataset while it is being collected. A pragmatic approach for the flat field correction of a new dataset could thus be to use the average and sigma images of the last few thousand images collected of similar samples with any given camera. A further *a posteriori* correction may then also serve as a diagnostic tool to highlight new flaws arising in the image transducer or in the *a priori* data normalization.

In one example the average and sigma images from two different datasets, collected on the same camera but almost a year apart, revealed that: some old dust particles remained in the same position on the chip; new “dust” particles were deposited; and some old dust particles had moved to a different location (Supplementary Fig. S7). Also in conventional photography, dust collection on the sensor of cameras with interchangeable lenses is a recurring problem. Software dust removal is often implemented in professional cameras or in post-processing software. In these cases an input image is typically required of a homogeneous featureless area like a white wall for the dust removal. Whereas such a correction will visibly reduce the influence of new dirt on the sensor, it cannot correct for the sensitivity loss suffered by pixels (partly) covered by the dirt on the sensor as does our proposed *a posteriori* correction. Finally, the normalization images derived from large datasets, i.e. the average and sigma images, characterize the state of health of the sensor at the time of data collection. This emphasizes the importance of long-term storage of raw datasets for quality control and validation purposes.

For many tasks in scientific image processing, the routine *a priori* correction of the image transducer properties is insufficient. This can be due to flaws of the image transducer or flaws of the flat-field correction applied, but can also be a consequence of the limited number of grey values available in the corrected image and/or of the limited time allocated for measuring the transducer characteristics. We have shown that we can determine the characteristics of the digital imaging sensor directly from large datasets, allowing us to perform *a posteriori* data corrections that are matched to the experimental conditions. The statistical independence of corrected images can readily be verified by Fourier Ring Correlation. The reduction of the fixed-pattern noise in the datasets leads to an overall improvement of the signal-to-noise ratio implying that more can be achieved with less data. The approach is simple to integrate in data-collection routines and leads to consistent datasets with a significantly reduced level of artefacts.

## Methods

The *a posteriori* determination of sensor characteristics and subsequent normalization of the data are based on the availability of a large dataset collected with the same camera under the same circumstances. For simplicity, we assume that basic errors in the image transducer such as missing pixels or row/columns (“dead” and “hot” pixels) have been corrected by a reasonable *a priori* correction procedure that a user does not normally have access to. This correction procedure will ensure that none of the pixels of the sensor will always produce the same numerical output value, thus avoiding division-by-zero problems in our *a posteriori* correction (equation (1)).

A further assumption is that the large dataset is homogeneous in a statistical sense (unimodal distributions of intensities and their standard deviations) and is not an agglomeration of diverse types of data collection on the same sensor. For example, an automatically collected cryo-EM dataset will typically also contain a number of entirely blank images or other deviant images which disturb the overall statistics. Such images are easily discarded after studying histograms of the average densities and/or the standard deviations of the individual images.

We also assume a homogeneous distribution of objects over the area of the images such that the chance of having a certain contrast at a certain position is isotropically distributed. The images should, for example, preferably not all have the same basic motif - such as a horizon running through the middle of the image with a bright sky above and a dark earth below the horizon. This issue was relevant in the processing of the differential interference contrast microscopy images (see Supplementary Fig. S5). Another implicit assumption is that the illumination is uniform over the field of view of the imaging sensor. When that is not the case, say a microscope with a misaligned illumination, then the resulting density ramp will be interpreted as a sensitivity ramp of the sensor and the images corrected accordingly (see Supplementary Fig. S5).

The *a posteriori* correction for the more general case than as formulated above (equation (1)), now including a target standard deviation 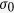 and a target average dataset density 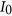, has the following form:


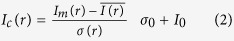


where the corrected image intensity 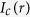 is derived from the measured 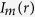 raw image, by subtracting 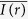, the average image over the full large dataset, normalized by the standard deviation image of that dataset 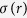. Here the original contrast of the data is restored by multiplying the results by average standard deviation of all pixel vectors 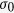, and adding the original average density 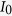 to all output images. Equation (2) can, but need not, be interpreted as dimensionless.

As discussed above, the FRC is used to identify spurious correlations between two images in the dataset. However, when those two images have also been used to calculate the average 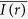 and sigma 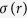 images that were used for the correction (as part of the full dataset), that procedure can introduce unintended correlations in the FRC curve. For smaller datasets in particular, to avoid such artificial correlations, it is thus best to correct the images to be used for FRC tests with an average and a sigma image to which they have not contributed.

A dedicated program (‘camera_norm’) for *a posteriori* correction was developed in the context of the IMAGIC 4D software system^23^,^24^. Complementing the examples given in the main paper, further examples from different fields are presented in the Supplementary Information.

## Additional Information

**How to cite this article**: Afanasyev, P. *et al*. *A posteriori* correction of camera characteristics from large image data sets. *Sci. Rep.* **5**, 10317; doi: 10.1038/srep10317 (2015).

## Supplementary Material

Supplementary Information

## References

[b1] Boyle W. S. & Smith G. E. Charge coupled semiconductor devices. At & T Tech. J. 49, 587 -+ (1970).

[b2] Aikens R. S., Agard D. A. & Sedat J. W. Solid-state imagers for microscopy. Method Cell Biol. 29, 291–313 (1989). 10.1016/s0091-679x(08)60199-5 2643764

[b3] Yu X., Jin L. & Zhou Z. H. 3.88 A structure of cytoplasmic polyhedrosis virus by cryo-electron microscopy. Nature 453, 415–419 (2008). 18449192 10.1038/nature06893 PMC2746981

[b4] Campbell M. G. *et al.* Movies of ice-embedded particles enhance resolution in electron cryo-microscopy. Structure 20, 1823–1828 (2012). 23022349 10.1016/j.str.2012.08.026 PMC3510009

[b5] Li X. *et al.* Electron counting and beam-induced motion correction enable near-atomic-resolution single-particle cryo-EM. Nature Meth. 10, 584–590 (2013). 10.1038/nmeth.2472 PMC3684049 23644547

[b6] Bai X. C., Fernandez I. S., McMullan G. & Scheres S. H. Ribosome structures to near-atomic resolution from thirty thousand cryo-EM particles. eLife 2, e00461 (2013). 23427024 10.7554/eLife.00461 PMC3576727

[b7] Amunts A. *et al.* Structure of the yeast mitochondrial large ribosomal subunit. Science 343, 1485–1489 (2014). 24675956 10.1126/science.1249410 PMC4046073

[b8] Kuhlbrandt W. Biochemistry. The resolution revolution. Science 343, 1443–1444 (2014). 24675944 10.1126/science.1251652

[b9] Kunath W., Weiss K., Sackkongehl H., Kessel M. & Zeitler E. Time-resolved low-dose microscopy of glutamine-synthetase molecules. Ultramicroscopy 13, 241–252 (1984). 6148794 10.1016/0304-3991(84)90203-1

[b10] Borland L. & van Heel M. Classification of image data in conjugate representation spaces. J. Opt. Soc. Am. A 7, 601–610 (1990).

[b11] van Heel M., Keegstra W., Schutter W. & van Bruggen E. in The Structure and Function of Invertebrate Respiratory Proteins (ed Wood E. J.) “Arthropod hemocyanin structures studied by image analysis”, 69–73 (EMBO 1982).

[b12] Saxton W. O. & Baumeister W. The correlation averaging of a regularly arranged bacterial-cell envelope protein. J. Microsc-Oxford 127, 127–138 (1982). 10.1111/j.1365-2818.1982.tb00405.x 7120365

[b13] Harauz G. & van Heel M. Exact filters for general geometry three dimensional reconstruction. Optik 73, 146–156 (1986).

[b14] van Heel M. Similarity measures between images. Ultramicroscopy 21, 95–99 (1987).

[b15] van Heel M. & Schatz M. Fourier shell correlation threshold criteria. J. Struct. Biol. 151, 250–262 (2005). 16125414 10.1016/j.jsb.2005.05.009

[b16] Vila-Comamala J. *et al.* Characterization of high-resolution diffractive X-ray optics by ptychographic coherent diffractive imaging. Opt. Express 19, 21333–21344 (2011). 22108984 10.1364/OE.19.021333

[b17] Karplus P. A. & Diederichs K. Linking crystallographic model and data quality. Science 336, 1030–1033 (2012). 22628654 10.1126/science.1218231 PMC3457925

[b18] Nieuwenhuizen R. P. *et al.* Measuring image resolution in optical nanoscopy. Nature Meth. 10, 557–562 (2013). 10.1038/nmeth.2448 PMC4149789 23624665

[b19] Banterle N., Bui K. H., Lemke E. A. & Beck M. Fourier ring correlation as a resolution criterion for super-resolution microscopy. J. Struct. Biol. 183, 363–367 (2013). 23684965 10.1016/j.jsb.2013.05.004

[b20] Nejadasl F. K., Karuppasamy M., Koster A. J. & Ravelli R. B. G. Defocus estimation from stroboscopic cryo-electron microscopy data. Ultramicroscopy 111, 1592–1598 (2011). 21945999 10.1016/j.ultramic.2011.08.007

[b21] Brilot A. F. *et al.* Beam-induced motion of vitrified specimen on holey carbon film. J. Struct. Biol. 177, 630–637 (2012). 22366277 10.1016/j.jsb.2012.02.003 PMC3322646

[b22] Vulovic M., Rieger B., van Vliet L. J., Koster A. J. & Ravelli R. B. A toolkit for the characterization of CCD cameras for transmission electron microscopy. Acta Cryst. D 66, 97–109 (2010). 20057054 10.1107/S0907444909031205

[b23] van Heel M., Harauz G., Orlova E. V., Schmidt R. & Schatz M. A new generation of the IMAGIC image processing system. J. Struct. Biol. 116, 17–24 (1996). 8742718 10.1006/jsbi.1996.0004

[b24] van Heel M. *et al.* Four-dimensional cryo electron microscopy at quasi atomic resolution: IMAGIC 4D. Int. Tab. Cryst. **F**, 624–628 (2012) doi:10.1107/97809553602060000875.

