# Supplementary material for: A posteriori correction of camera characteristics from large image data sets
Source: Sci Rep. 2015 Jun 11;5:10317. doi: 10.1038/srep10317 (PMC4464200; doi:10.1038/srep10317)
Supplement: Supplementary Information [file srep10317-s1.pdf]

Supplementary Information

A posteriori correction of camera characteristics from large image data sets

Pavel Afanasyev, Raimond B.G. Ravelli, Rishi Matadeen, Sacha De Carlo,

Gijs van Duinen, Bart Alewijnse, Peter J. Peters, Jan-Pieter Abrahams,

Rodrigo V. Portugal, Michael Schatz, Marin van Heel

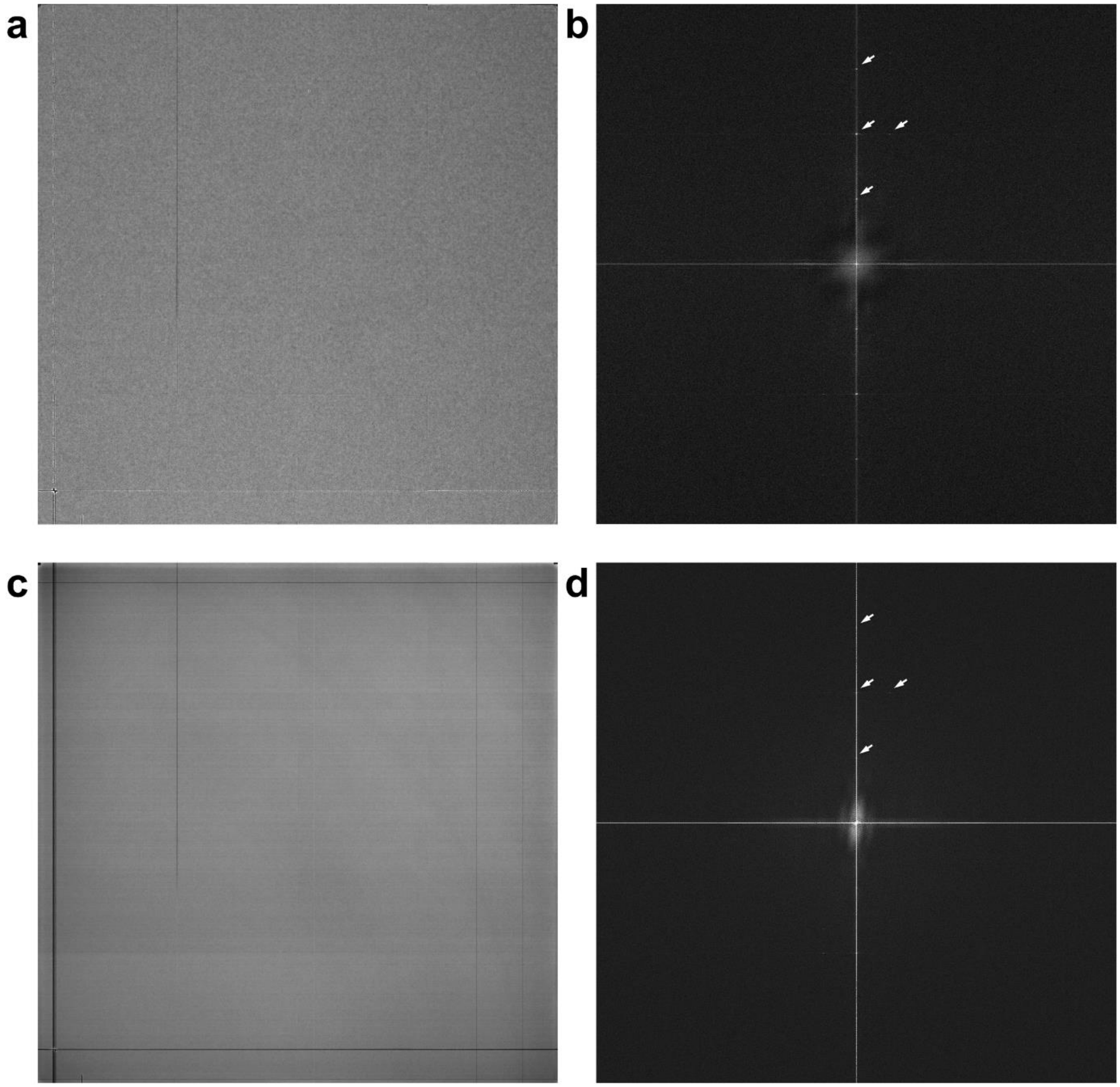

Supplementary Figure S1 | Fourier Spectra. (a) The average of the full dataset of 70S ribosome (Fig. 1 of the main paper). This average image is of full size (4096x4096 pixels); note that the lower left corner of (a) is shown in detail in Figure 1c of the main paper. The amplitude spectrum of the full-scale average is shown in (b). Highlighted by arrows in this spectrum are some peaks associated with the electronics of the camera leading to spurious peaks at ¼, ½ and ¾ of the Nyquist frequency. A further spurious peak is visible at ½ Nyquist in the vertical direction yet slightly offset to the right. These systematic peaks in the 2D amplitude spectra lead to the peaks revealed in the FRC calculation (main paper Fig. 2). The sigma image of the full dataset (c) reveals the flawed areas of this experimental sensor. Its amplitude spectrum (d) shows similar features as does the total-average amplitude spectrum (b). Note that after the *a posteriori* correction of a large dataset their new average image and sigma image both become constants.

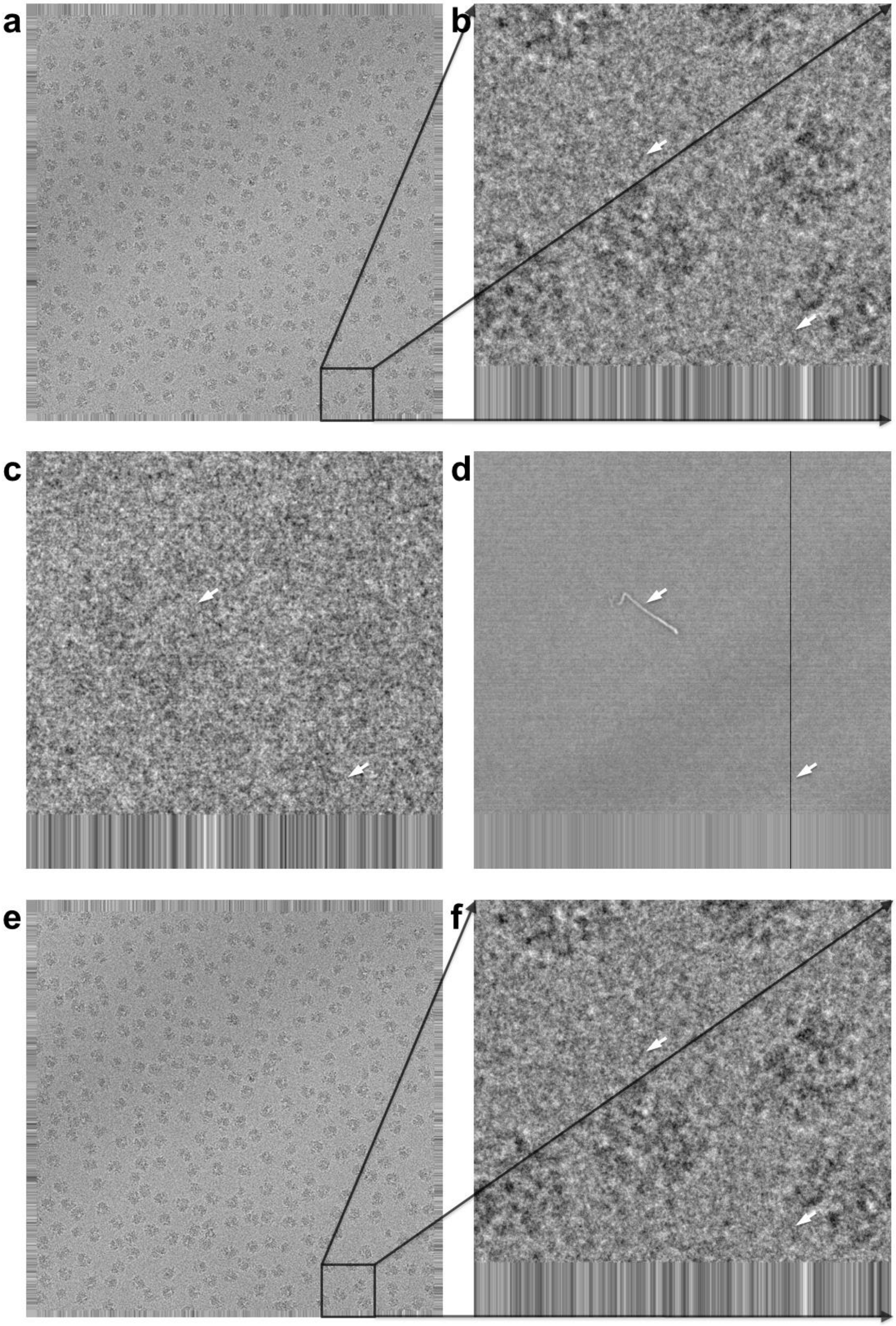
a
b
c
d
e
f

Supplementary Figure S2 | *A posteriori* correction of cryo-EM dataset of *S. cereviseae* 80S ribosomes. (a) A typical 4096x4096 pixel back-thinned FEI Falcon direct electron detector image from the publically available Electron Microscopy Pilot Image ARchive (EMPIAR) (http://pdbe.org/empiar; dataset EMPIAR-10002). The edges of the images each have a fixed repeating pattern (“apodization”) which pattern differs from image to image in the 4,160 frames of this entry (4,160 =260x16 movie frames). For the details of this dataset see Bai *et al.* 2013[1]. In (b), a 512x512 part of the lower edge of the first image (a) is shown in detail. Whereas the apodization of the edge is clearly visible, other anomalies such as dust particle and a missing vertical column remain invisible. In figure (c), the overall average of these 4160 512x512 sub-images is shown; two arrows mark the positions of two anomalies that have apparently removed by *a priori* correction but are clearly visible in the standard deviation (sub-) image (d). The *a posteriori* corrected images are shown in (e) and the detail area in (f).

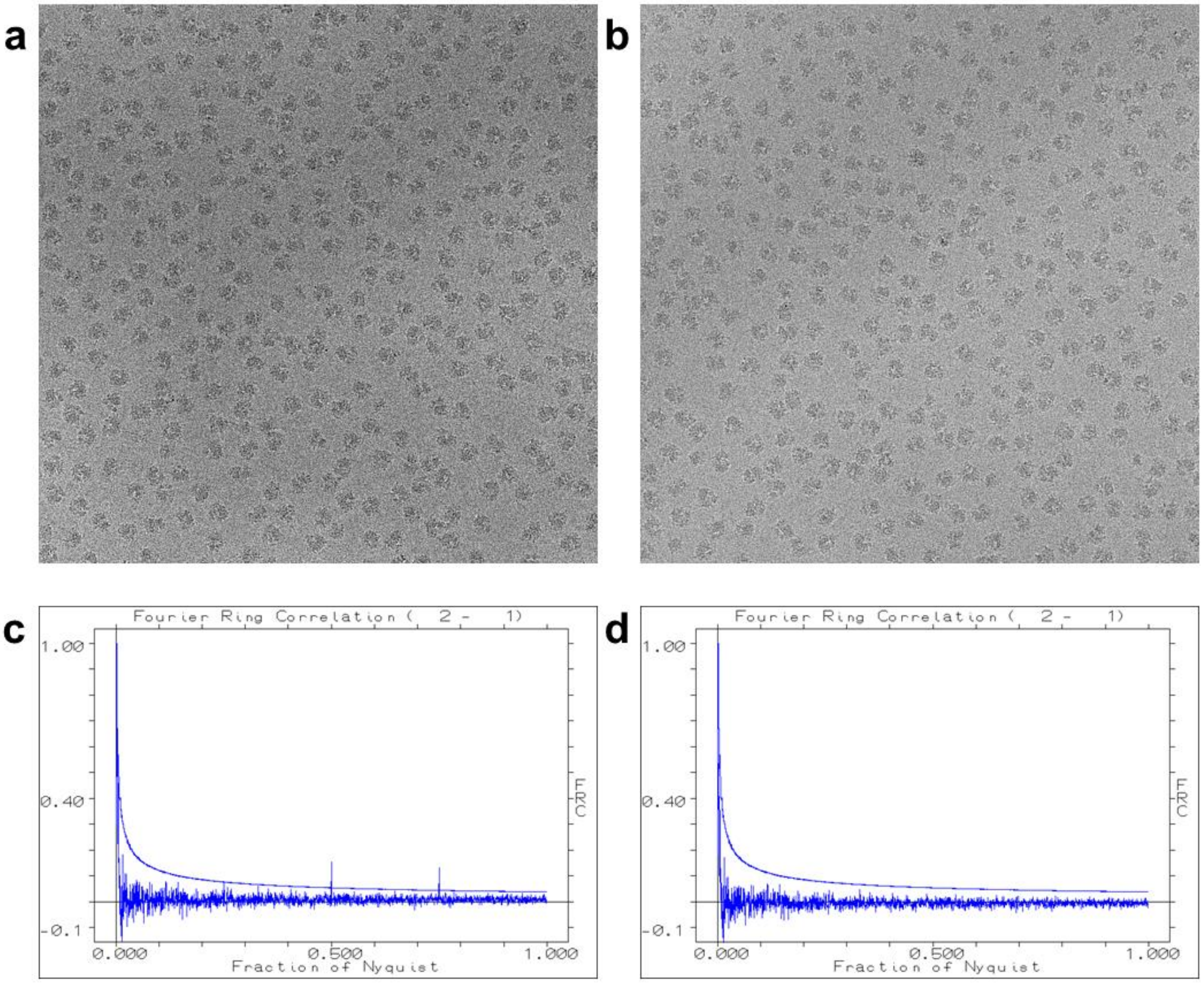

Supplementary Figure S3 | Fourier Ring Correlation (FRC) of two ribosome micrographs. Two typical 3888 x 3888 sub-images of the 4096x4096 pixel images from the dataset shown/discussed in Supplementary Fig. 2. The *a priori* correction that had been applied to these images (Supplementary Fig. 2) was already quite good in providing uncorrelated images as exemplified in (c) which shows the FRC between images (a) and (b). The *a posteriori* correction (d) succeeded in removing the remaining spurious correlation peaks at 0.5 and 0.75 Nyquist frequency that are correlated to the read-out electronics of the sensor. The sensor in this case was an experimental sensor back-thinned direct electron detector, comparable to the sensor used for generating the Figure 1 dataset.

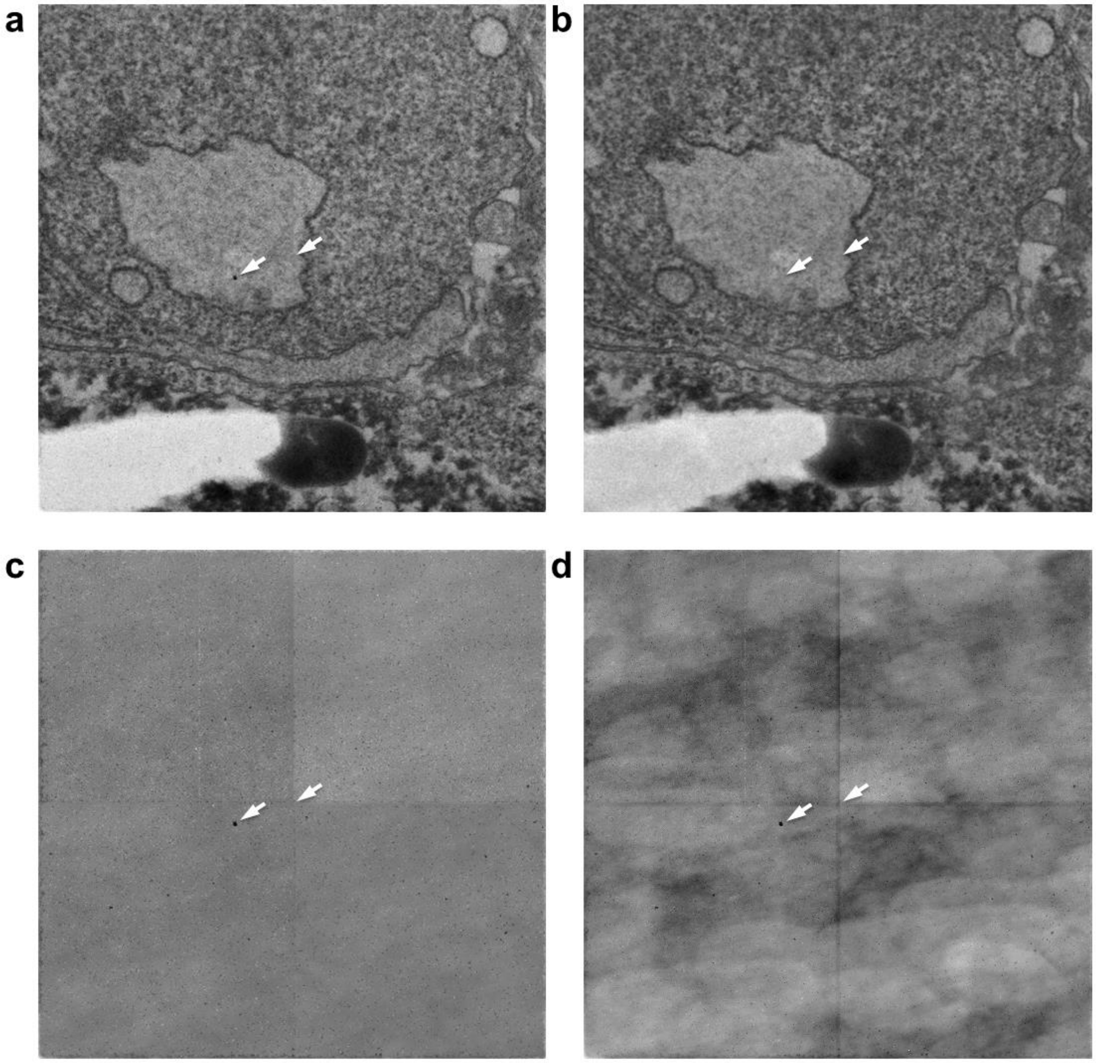

Supplementary Figure S4 | *A posteriori* correction of a Medipix camera dataset.
A Medipix2 camera[2, 3] was used to acquire a dataset of stained cell sections consisting of 684 transmission electron microscopy images of each 512x512 pixels. The detector of this Medipix2 camera consists of 4 tiled sensors of 256x256 pixels each. The gaps between the four sensors cause a visible cross in the middle of the 512x512 image. The gaps were corrected for by an *a priori* correction[2] just after data acquisition but remain clearly visible as a blurred band in the raw images (a). In contrast to the other tests included in this publication we here had full access to the raw data since the *a priori* correction software was written and applied by one of us (JPA). For consistency, however, we fully ignored our specific knowledge of the details of this pre-processing algorithms. The *a priori* corrected images were thus *a posteriori* corrected (b). The artificial cross in the middle of the images and the many dead - and hot pixel artefacts as well an anomalous (dotted-line) column disappeared after that correction. These anomalies were all clearly visible in the average image (c) and in the standard deviation image (d). (Information on the Medipix2 camera can be found at the following website: http://medipix.web.cern.ch /medipix/).

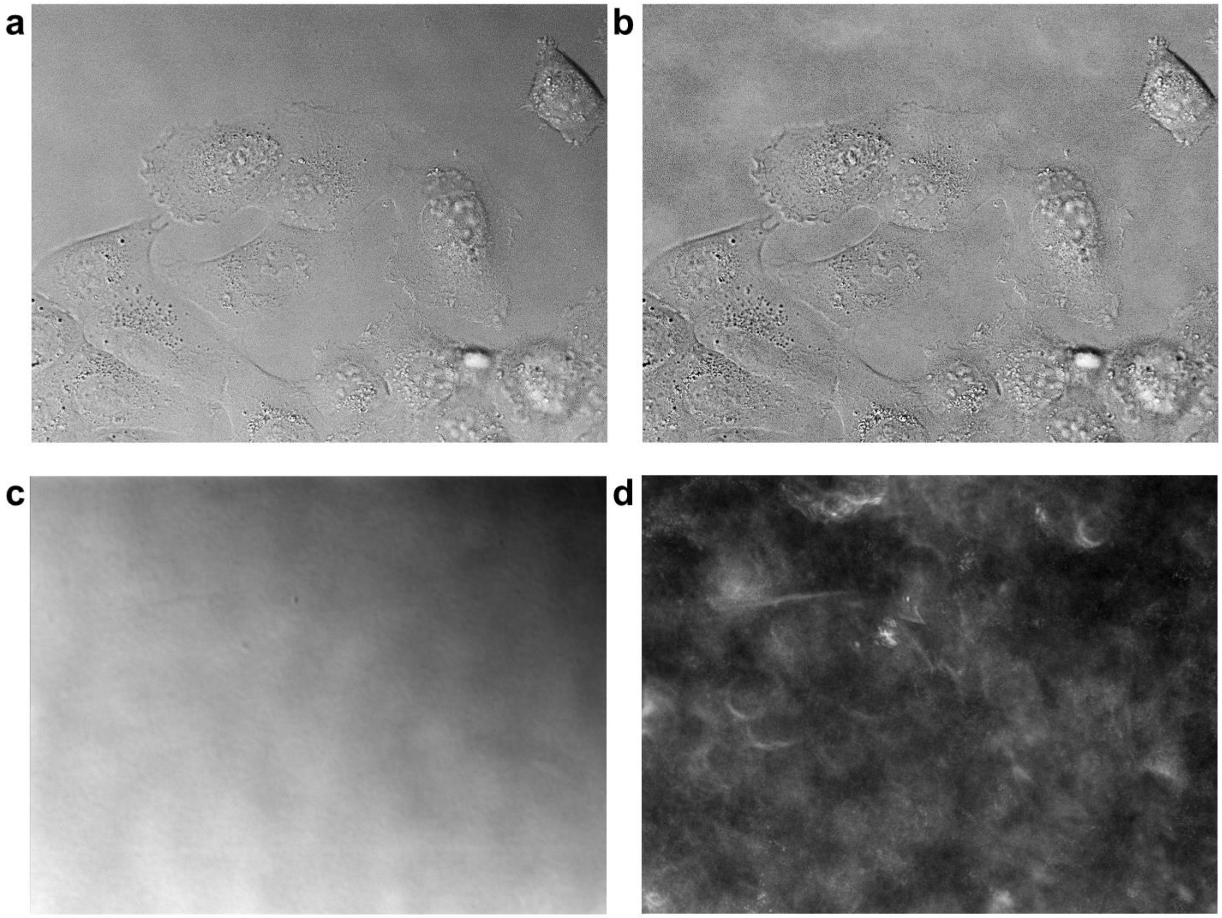

Supplementary Figure S5 | Differential interference contrast microscopy (DIC). This dataset was collected during an overnight live cell imaging experiment, and consists of 2,501 images (a) of 1344 x 1024 pixels each, acquired on a Hamamatsu ORCA-R2 CCD camera. The average of the dataset shows an uneven illumination with a ~25% density gradient in a diagonal direction over the full field of view (c). This suggests that the illumination of the microscope was misaligned. The standard deviation image of the dataset (d) has residual patterns from the original image information in the dataset. In spite of the relatively large size of the dataset (2,501 images, remaining after excluding more than 1,000 images with a strongly differing standard deviation), the movement of the cells within the image frame were slow leading to specific areas exerting more contrast than others. The bright spots in the sigma images can be related to specific high-contrast objects being imaged which persist in specific places over prolonged periods of time. The removal of the background ramp in the corrected image (b) allows one to use the full available density-range for visualizing the object details in full contrast.

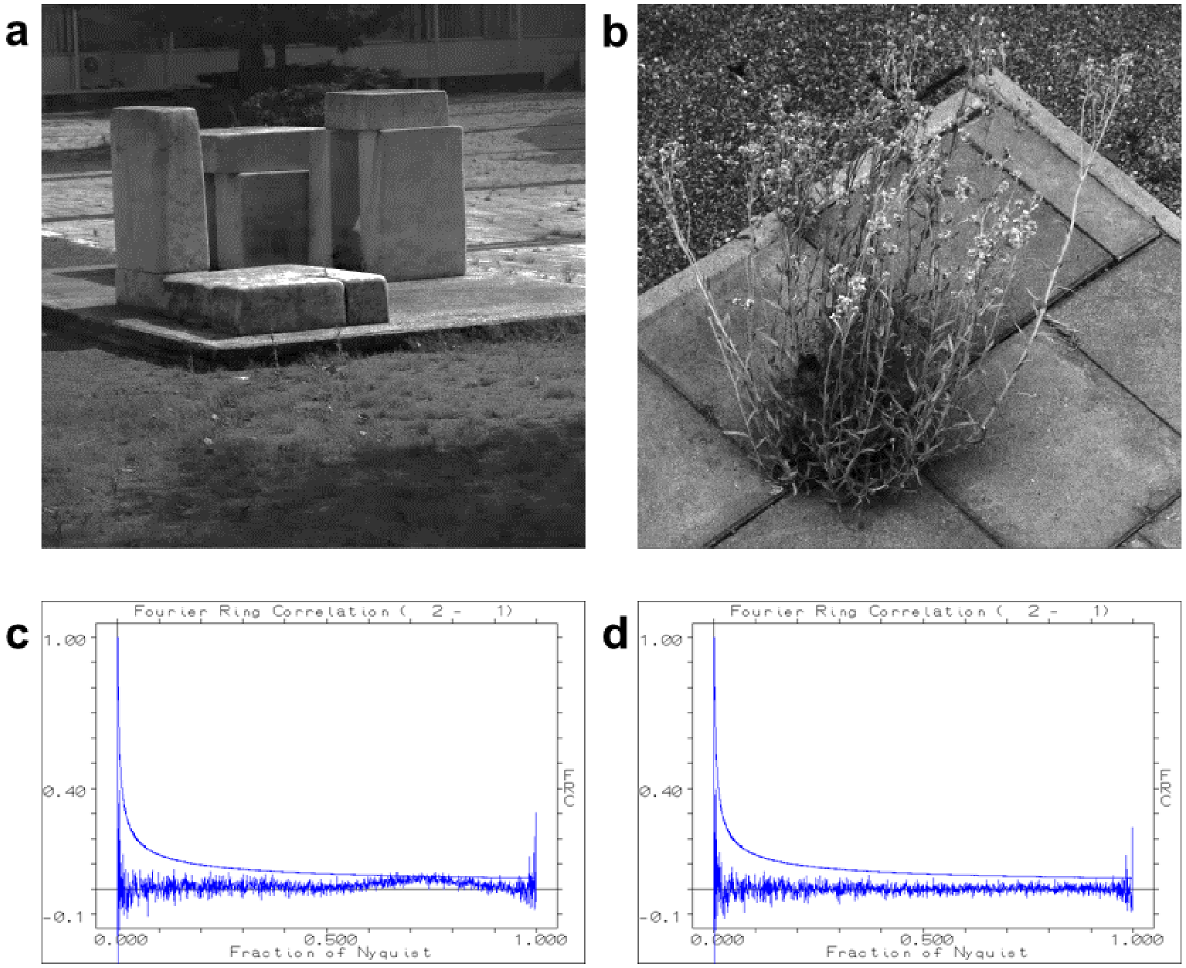

Supplementary Figure S6 | Standard Digital Photography. A Sony Alpha A350 consumer DSLR-camera was used to acquire a dataset of 666 RGB images 3072x4600 in ARW format. These images were converted to an uncompressed TIFF format treating each pixel as a separate grey-level measurement using LibRaw decoder (unprocessed_raw utility http://www.libraw.org/) followed by conversion into IMAGIC format (https://www.imagescience.de/formats) using the em2em converter (https://www.imagescience.de/em2em). The results are therefore fully comparable to the images produced by the Mastcam-right camera of the Mars rover (Fig. 3). Two typical images (a) and (b) taken from the Sony camera dataset are compared to each other by FRC before (c) and after (d) *a posteriori* correction. These FRC curves indicate that the images correlate less at high resolution (~0.5 Nyquist and beyond) than do the Curiosity mars-rover images (Fig. 3, 4). This is probably due to the physical low-pass filter placed in front of the sensor in the A350 camera which removes high-resolution information prior to the image acquisition. Such a filter is not present in scientific grade sensors as used in the Mars rover camera.

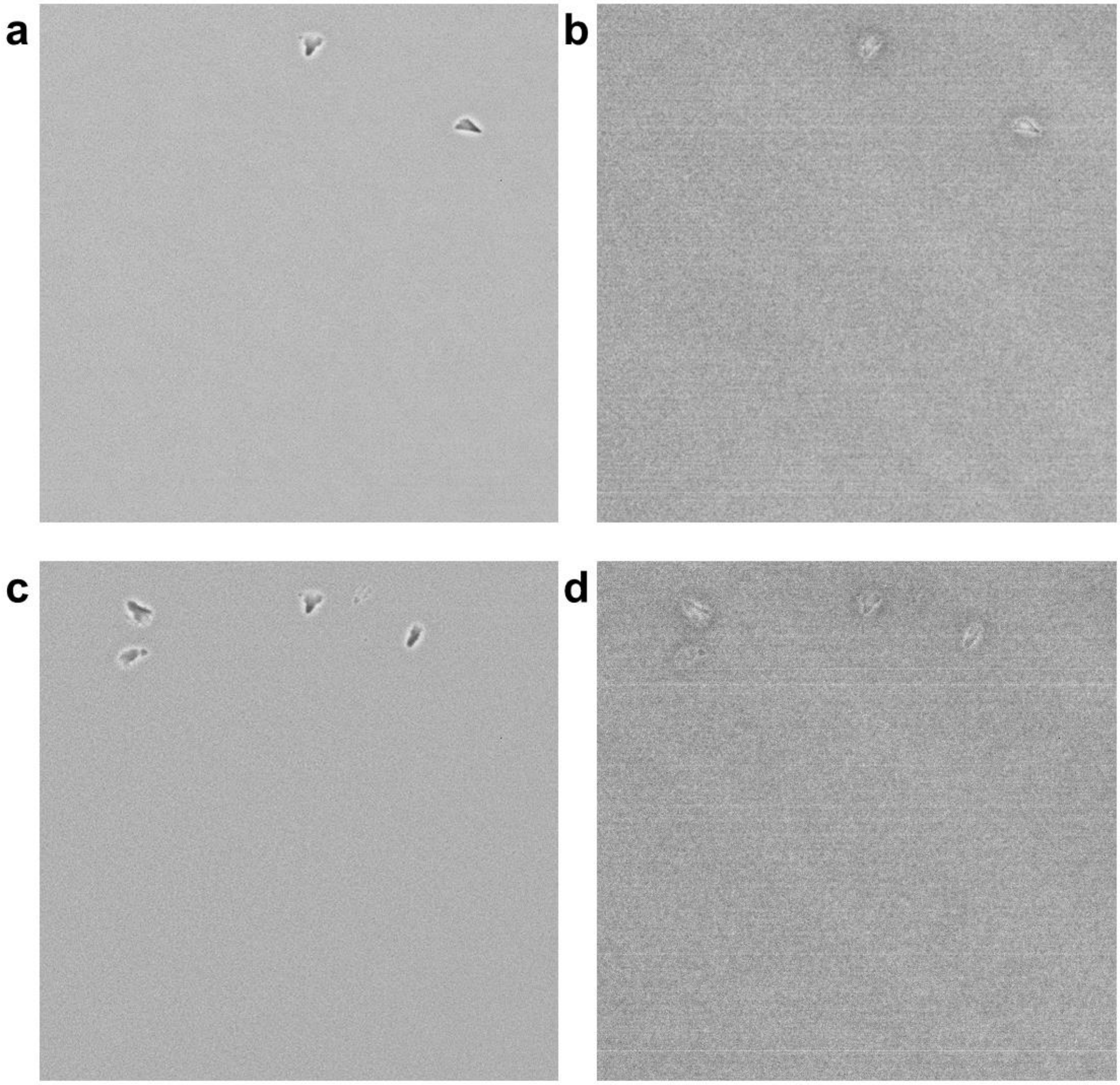

Supplementary Figure S7 | Dust changes over time. Two different cryo-EM datasets, collected on the same sensor but about a year apart, illustrate changes in "Dust Accumulation". Figures (a) and (b) show the average and the standard deviation images of dataset "one" collected in May 2012 on an FEI Falcon-1 direct electron detector. Similarly (c) and (d) show the average and the standard deviation images of dataset "two" collected in March 2013 on the same microscope and sensor. (All images are of the same 512x512 detailed area extracted from the full 4096x4096 images). The same "dust" particles can often be detected at the same position in the later dataset but some dust particles have changed position and/or orientation. New dust particles have appeared on the sensor after a year of use that were not seen in the earlier images.

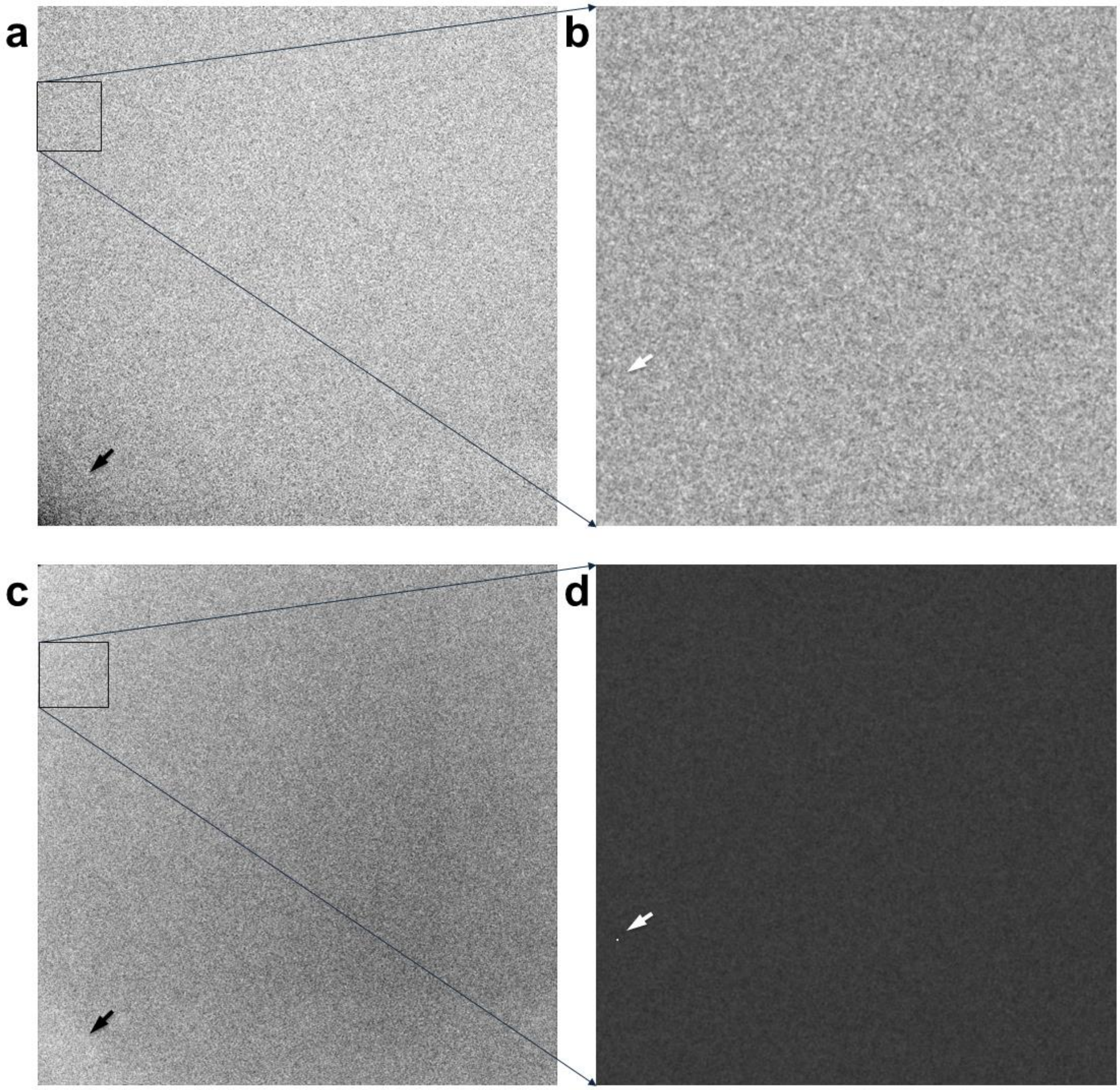

Supplementary Figure S8 | Dataset, collected manually over three consecutive days on a TVIPS F416 CMOS camera, contains 1,164 images. A significant number of images 174 were discarded based on the histograms of their individual statistics (average densities and standard deviations). This was due to the dataset being mixed with overview images and due to known instabilities in the camera electronics rendering some images unusable (the camera was already scheduled for replacement). The overall average image (a) has a slightly lower density in the lower–left corner (marked by a black arrow) yet that area also has a higher gain that the rest of the image (c). A 512x512 detail from the average image marked with a black square in (a) is shown in (b), revealing a very homogeneous density response in that patch. The corresponding σ-image 512x512 detail (d), in contrast, reveals the presence of a deviant pixel with an extreme standard deviation behaviour.

References

1. Bai, X.C., Fernandez, I.S., McMullan, G. & Scheres, S.H. Ribosome structures to near-atomic resolution from thirty thousand cryo-EM particles. *eLife* **2**, e00461 (2013).

2. Nederlof, I., van Genderen, E., Li, Y.W. & Abrahams, J.P. A Medipix quantum area detector allows rotation electron diffraction data collection from submicrometre three-dimensional protein crystals. *Acta Cryst. D* **69**, 1223-1230 (2013).

3. Llopart, X., Campbell, M., Dinapoli, R., SanSegundo, D. & Pernigotti, E. MEDIPIX2: A 64-k pixel readout chip with 55^{1}m2 elements working in single photon counting mode. *Trans. Nucl. Sci.* **49**, 2279-2283 (2002).
